# Mid-Crisis Restructuring Law Reform in the United Kingdom

**DOI:** 10.1007/s40804-023-00288-0

**Published:** 2023-05-04

**Authors:** Kristin van Zwieten

**Affiliations:** 1 Clifford Chance Professor of Law and Finance, Law Faculty, University of Oxford, Oxford, UK; 2 Gullifer Fellow, Harris Manchester College, Oxford, UK; 3 Research Member, ECGI, Brussels, Belgium

**Keywords:** Restructuring law, Insolvency law, Insolvency law reform, Restructuring law reform, Emergency rule-making, Covid-19

## Abstract

Economic shocks create insolvency law-making space, generating opportunities for legal reform that may be absent in good times. Policymakers may suddenly acquire a mandate to resource institutions or drive through a change in the law where in good times such reforms were likely to be foiled by interest group capture, or simply unlikely to get sufficient political traction. A crisis, then, is an opportunity for the well-prepared insolvency policymaker. Insolvency rule-making in crisis conditions is, however, plainly also risky. Making best use of the opportunity implies making more than temporary changes to the regime. But design choices made mid-crisis will almost inevitably be influenced by the features of the crisis itself, generating a risk that the result of the reform effort will be distorted law, ill-suited to the achievement of the lawmaker’s objectives in the long run. This paper considers the permanent restructuring law reforms enacted in the UK during the first wave of the Covid-19 pandemic. At first glance, these reforms appear to exemplify the case of the well-prepared policymaker, poised to drive through carefully planned changes to the law when the opportunity arises. On closer inspection, however, a different picture emerges. The permanent measures, which were enacted in a fast-track legislative process, departed from the Government’s pre-pandemic plan in material and undesirable ways. In some cases, these deviations mean that the original objective has not been achieved at all; in others, the objective has been at least partially achieved, but at unnecessary cost. Overall, the UK experience appears to better exemplify the risks of attempting insolvency law reform in a crisis, than the opportunities that a crisis affords to an insolvency policymaker.

## Introduction

Economic shocks create insolvency law-making space, generating opportunities for legal reform that may be absent in good times. Policymakers may suddenly acquire a mandate to invest in resourcing or creating new institutions (e.g., specialist courts) for the treatment of an expected surge in cases. Licence may be given to drive through a change in the substantive law, and/or in practice and procedure, where in good times such reforms were likely to be foiled by interest group capture,^1^ or were simply unlikely to get sufficient political traction. A crisis, then, is an opportunity for the well-prepared policymaker; that is, one who has already worked to understand how the existing regime functions, and how it might be improved, and identified their preferred solution. As the OECD observed in the first year of the Covid-19 pandemic: ‘This crisis can provide an opportunity for [insolvency] reforms which are often hard to implement and take time to design’.^2^

If, however, the lawmaker’s ultimate objective is to deliver an insolvency regime that is conducive to investment in good times as well as in bad, insolvency rule-making in a crisis is plainly also risky. Making best use of the opportunity implies making more than merely temporary changes to the regime. But any design choice made mid-crisis will almost inevitably be influenced by the features of the crisis itself, since the reformer’s mandate is grounded in the need to make better provision for the treatment of crisis-affected firms. There is, therefore, a plain risk that the regime will be permanently changed in ways that avoid the magnification of the shock in the short run, but increase financial constraints in the long run. So, for example, a crisis reformer might focus on ensuring the availability of a restructuring procedure that would shield newly distressed businesses from forced sales to third parties^3^ (which might otherwise amplify the shock^4^), but neglect to consider how such a procedure will operate in the post-crisis period, when it ought to be simpler to distinguish viable from non-viable businesses, and will be essential to exclude the latter from procedures that would merely function to delay a wind-down of the business at creditor expense. A failure to do so would be expected to tighten financial constraints *ex ante*.^5^

In normal times, the law reform process might reveal such unintended consequences and/or the availability of lower-cost routes to achieving the reformer’s objective. This is the function of a regulatory impact assessment, a process in which the reformer is forced to (publicly) articulate the parameters of the problem to which the proposed reform responds, identify plausible routes to responding to that problem, articulate the expected costs and benefits of each, and weigh these against the baseline (‘do nothing’).^6^ In a parliamentary democracy, this is also a function of the ordinary legislative process, in which there can be ‘line-by-line’ scrutiny of draft legislation, having regard to feedback received from constituents and/or interest groups.^7^ In emergencies, however, governments truncate the ordinary legislative process and carry out ‘lighter touch’ consultation (notice that this may reinforce a tendency to tailor the contours of the reform to reflect the peculiar features of the crisis^8^), promising a full evaluation in a later period.^9^ But by the time such evaluation occurs, which will almost inevitably be in a post-crisis period, there may no longer be any (political) opportunity to alter course. By that time, the pre-crisis regime has already been altered, and is in the process of being ‘bedded in’ by the relevant professional and adjudicatory bodies.

This paper focuses on the insolvency reforms made in the UK by the Corporate Insolvency and Governance Act 2020 (hereinafter ‘CIGA’). CIGA was enacted in a fast-track process early in the Covid-19 pandemic, with a promise of review within three years of commencement.^10^ CIGA temporarily modified pre-pandemic insolvency law to make it more difficult for creditors to force an insolvent company into liquidation,^11^ and to reduce the risk of personal liability for directors in keeping companies outside formal insolvency proceedings.^12^ But the Act also permanently changed the law in radical ways; not since the Government’s landmark reforms in 2002 has such a reorientation of the legal framework been attempted.

In 2002, the long-established administrative receivership^13^ procedure was largely^14^ removed to make the administration procedure the main alternative to liquidation for insolvent companies. Administration had been on the statute book since 1985, but lenders with global security had been empowered to pre-empt the making of an administration order^15^ by appointing an administrative receiver to act in their (the secured lender’s) interests.^16^ The 2002 reforms prospectively^17^ abolished administrative receivership for most categories of case, made it possible for administration to be commenced out-of-court,^18^ and directed the administrator to act in the interests of the general body of unsecured creditors.^19^

Although administration was presented as a ‘rescue’ alternative to liquidation,^20^ it was better suited to enabling a sale of the business to a third party (‘business rescue’) than to enabling the preservation of the debtor itself through a corporate reorganisation (‘corporate rescue’). The insolvency legislation (the Insolvency Act 1986) itself made only limited provision for binding dissenting creditors to a restructuring plan (through a ‘company voluntary arrangement’),^21^ and the procedure available under the Companies Act 2006 for doing so (the ‘scheme of arrangement’) was limited in other ways. Restructuring professionals worked creatively with what they had,^22^ but these solutions had limits, and their own costs. The permanent changes made in 2020 by CIGA were aimed at filling gaps in the ‘restructuring toolkit’^23^ with a view to facilitating corporate rescue.

CIGA added a new restructuring procedure to the Companies Act 2006,^24^ the ‘restructuring plan’, in which (unlike in a scheme of arrangement) dissenting classes of creditors (or members) can be bound to a plan. CIGA also made two significant additions to the Insolvency Act 1986: a ‘freestanding’, debtor-in-possession, moratorium now sits alongside the (director-displacing) administration and liquidation procedures, and contracts for the supply of goods and services to a debtor in proceedings under the Act (or for whom a meeting is summoned for voting on a restructuring plan) are now regulated with a view to ensuring continuity of supply.^25^ Under the old law, there were few constraints on the exercise of rights to terminate an executory contract on the counterparty’s entry into insolvency proceedings (‘*ipso facto* clauses’).^26^

It was feasible for such significant changes to be made in a fast-track legislative process because the Government had committed to making changes of these kinds prior to the pandemic, having consulted extensively (with impact assessments) on doing so. A freestanding moratorium was proposed in 2009.^27^ In 2016, the Government added proposals for some constraints on *ipso facto* clauses in contracts for supply, and for a new ‘multi-class restructuring procedure’.^28^ The Government committed to implementing a form of these proposals in 2018,^29^ but there was no legislative activity until the onset of the pandemic. On 28 March 2020, the Government promised legislation ‘at the earliest opportunity’,^30^ and the Corporate Insolvency and Governance Bill was introduced to Parliament on 20 May 2020, accompanied by an impact assessment that drew on analysis from the pre-pandemic consultations. In the first debate on the Bill, the Business Secretary was quick to emphasise that the permanent measures had been consulted on in detail prior to the pandemic.^31^

At first glance, then, CIGA appears to exemplify the case of a well-prepared policymaker, poised to drive through carefully planned changes to the law when the opportunity arises. The fact that the Act temporarily modified two of the three permanent measures in a bid to tailor them to pandemic conditions^32^ lends support to this analysis. On closer inspection, however, a different picture emerges. Close analysis of the detail of the permanent measures suggests that the emergency context affected their design, either directly (as where a design choice is difficult to understand other than by reference to the pandemic), or indirectly (as where legislative provisions appeared to have suffered from being produced in a fast-track process). In relation to the moratorium, some design choices mean that the procedure is arguably unable to achieve the stated objective. In relation to contracts for supply, the objective was at least partially achieved, but arguably at unnecessary cost. In relation to the restructuring plan procedure, the decision to produce this in a truncated legislative process meant that the most difficult design choice (namely, the factors relevant to the exercise of discretion to sanction a plan in the presence of a dissenting class) was left for courts to make *ex post*.

In Section 2, I contextualise the CIGA reforms by introducing other aspects of the Government’s early response to Covid-19 business distress, which included restrictions on landlords’ ability to enforce the payment of rent, and a generous package of state-guaranteed loans (permitting businesses to borrow to meet fixed costs). It seems likely that a non-trivial proportion of these liabilities (new loans, deferred rent) will not be able to be repaid in full; if businesses are to remain in the hands of existing owners during the current downturn, some restructuring will be required. In Section 3, I turn to CIGA’s permanent restructuring measures. I explain the background to the Government’s decision to introduce a moratorium, a restructuring plan procedure, and constraints on the termination of contracts for supply. I then turn to the implementation of this plan through CIGA, identifying a series of undesirable departures from the Government’s pre-pandemic thinking, and further arguing that the rushed passage of the Bill meant that an opportunity was lost to consider how best to fit the pre-pandemic plan to the existing legal framework. Whilst problems in the drafting of each individual measure can, at least in theory, be cured, the latter opportunity is not recoverable. In Section 4, I turn briefly to the question of how we might have done better, with a view to informing analogous reform exercises in future crises.

## CIGA in Context: The Government’s Pandemic Relief Package for Businesses

In March 2020, the UK Government began announcing restrictions on movement and trade with a view to delaying the spread of Covid-19. At the same time, it began announcing relief for businesses, and as restrictions intensified, the relief package widened. By 20 March 2020, the Government had promised to provide cash grants to small businesses,^33^ reduce or abolish business rates for some businesses,^34^ assume 80% of the salary cost of employees kept on payroll up to £2,500 per month,^35^ defer the payment of VAT^36^ liabilities (tantamount to a ‘direct injection of £30bn of cash to employers, equivalent to 1.5% of GDP’^37^) and invited businesses to request the postponement of other tax liabilities, and to make state-guaranteed business loans available on an interest-free basis.^38^ A fund for the purchase of 1-year maturity commercial paper from investment grade issuers making a material contribution to economic activity in the UK^39^ was also announced.

In the week that followed, during which time households were issued with a ‘stay at home’ instruction, a raft of additional legislative measures were promised, including a ‘ban on evictions for commercial tenants who miss rent payments’,^40^ the ‘temporary suspension of wrongful trading provisions for company directors, to remove the threat of personal liability during the pandemic’, and ‘measures to improve the insolvency system’ with the ‘overriding objective’ of helping ‘UK companies which need to undergo a financial rescue or restructuring process to keep trading’.^41^ This legislative response would include ‘new rules to make sure companies undergoing restructuring can continue to get hold of supplies and raw materials’.^42^ Three weeks later, the Government announced that additional measures were necessary ‘to protect UK high street from aggressive rent collection and closure’, explaining:

The majority of landlords and tenants are working well together to reach agreements on debt obligations, but some landlords have been putting tenants under undue pressure by using aggressive debt recovery tactics.^43^

The Government advised that landlords under financial pressure could make use of new state-guaranteed bank lending facilities, and promised to add to its legislative agenda: (i) a temporary restriction on the use of the commercial rent arrears recovery procedure; and (ii) a temporary restriction on creditors’ ability to force companies into liquidation on the basis of inability to pay debts.^44^

New bank loans, guaranteed by the state, were at the centre of the ‘first wave’ policy response. On 17 March, the Chancellor said:

I can announce today an unprecedented package of government-backed and guaranteed loans to support business to get through this. Today, I am making available an initial £330 billion of guarantees, equivalent to 15% of our GDP. That means any business who needs access to cash to pay their rent, their salaries, suppliers or purchase stock, will be able to access a government-backed loan or credit on attractive terms. And if demand is greater than the initial £330 billion I am making available today, I will go further and provide as much capacity as required. I said, ‘whatever it takes’, and I meant it.^45^

Although the Government soon formulated additional policies for the treatment of wage and rent liabilities—as explained above, liability for wages was to a large extent assumed by the state, and rent was arguably effectively postponed through the early ban on eviction and the additional measures announced to restrict rent collection^46^—other types of fixed costs were not treated so directly. Instead, the strategy appears to have been to nudge forbearance by banks in relation to the payment of pre-pandemic bank debt,^47^ and to make available new state-guaranteed bank loans that could be used by businesses to meet any other residual fixed costs once cash reserves were exhausted.^48^

In pursuance of this policy, changes to bank regulatory policy were rapidly announced, including the release of the countercyclical buffer (said to ‘reinforce the expectation’ of the Financial Policy Committee and the Prudential Regulation Committee of the Bank of England that ‘all elements of banks’ capital and liquidity buffers can be draw[n] down as necessary to support the economy’^49^), along with a scheme to make funding available to banks ‘with additional incentives for banks to support lending to SMEs’.^50^ The underlying assumption was that ‘although disruption arising from Covid-19 could be sharp and large, it should be temporary’.^51^ Some £77.15 billion was extended to businesses in three bank loan schemes—the ‘Coronavirus Business Interruption Loan Scheme’, the ‘Coronavirus Large Business Interruption Loan Scheme’, and the ‘Bounce Back Loan Scheme’—along with the purchase of £37 billion in commercial paper issued by 107 investment grade issuers. The latter scheme was later closed, firms repaying what had been borrowed under the scheme, with interest.^52^ But as at September 2022, the bulk of the bank loan facilities remain unpaid—7.4% of all facilities have been repaid in full, and 10.8% are in arrears or default. 78.1% of these facilities are reported to be ‘on schedule’,^53^ but this should be treated with some caution, given that the vast majority^54^ of these facilities are Bounce Back Loans, and in 2021 the Government offered to extend the term for repayment of these loans in the light of ‘extended disruption from Covid-19’.^55^ The Bounce Back Loan scheme targeted SMEs, offering loans of up to £50,000, initially interest free, that were 100% guaranteed by the state;^56^ one quarter of UK businesses received a Bounce Back Loan.^57^

There has been substantial criticism of the Government’s loan schemes, particularly in relation to fraud control. The National Audit Office investigated the Bounce Back Loan Scheme, concluding that ‘the government prioritised payment speed over almost all other aspects of value for money’ and that ‘[t]he impact of prioritising speed is apparent in the high levels of estimated fraud’.^58^ But even if fraud is put to one side, it would have been plausible to expect that a non-trivial proportion of Covid-19 loans, and of rolled-over rent,^59^ would not be able to be repaid in full, given that pandemic-related restrictions on trade and movement were, in the end, protracted. The situation has since been compounded by a significant worsening of economic conditions, such that an increase in corporate defaults is now expected. If businesses are to remain in the hands of current owners during the present downturn, rather than subjected to forced sales to third parties, debts will need to be restructured. If this cannot be achieved consensually, businesses will need to use the framework provided by law for imposing a compromise on creditors. This framework was permanently changed in significant ways by CIGA.

## CIGA’s Permanent Restructuring Reforms

A review of the extraneous materials relating to CIGA^60^ paints a picture of a Government that somehow managed to ‘have it both ways’. On the one hand, the Corporate Insolvency and Governance Bill 2020 was presented as a response to the Covid-19 emergency, which justified the exceptional use of a fast-track^61^ legislative process. On the other hand, the restructuring-related measures contained in the Bill were measures that the Government had previously decided would benefit businesses generally, having consulted on this, such that it was legitimate for them to be introduced on a permanent, rather than merely temporary, basis. Thus, the Bill’s restructuring reforms were at once a crisis measure, and not a crisis measure.^62^ In this section, I argue that the Government failed in its attempt to ‘have it both ways’, and instead produced permanent law that bears the mark of the crisis context in which it was made, and suffers from this. I begin by explaining the Government’s thinking in proposing a moratorium, a restructuring plan procedure, and constraints on *ipso facto* clauses in contracts for supply, before turning to the implementation of this plan through CIGA.

### The Pre-pandemic Plan

The Government’s plan for restructuring law reform was developed slowly, through extensive stakeholder engagement, and from a modest starting point. The first set of proposals appeared in 2009,^63^ against the backdrop of the recession that followed the Global Financial Crisis. The Government was reasonably confident in the corporate insolvency framework: it was ‘highly regarded by external commentators’,^64^ scoring well in the World Bank’s ‘Doing Business: Resolving Insolvency’ assessment,^65^ and ‘working well’ during the downturn.^66^ But engagement with stakeholders had suggested that some ‘targeted measures to further improve our rescue culture’ might be welcomed.^67^

The main proposal related to the company voluntary arrangement (CVA) procedure in Part I of the Insolvency Act 1986, by which company directors may make a proposal for an arrangement with creditors.^68^ The Government was keen to encourage use of the CVA procedure for restructurings.^69^ The concern was that, other than for small companies, no moratorium was supplied by the Insolvency Act 1986 to support a CVA proposal, meaning that companies needed to enter administration proceedings to obtain a moratorium.^70^ The Government proposed to extend the small company CVA moratorium (which was equivalent in scope to the administration moratorium, and initially for 28 days) to larger companies,^71^ and to empower the court to impose a moratorium of up to 3 months’ duration for companies that were or were likely to become unable to pay their debts, and for which there was a reasonable prospect of a CVA being approved by creditors.^72^ A second proposal related to post-commencement finance: in response to reports from stakeholders of difficulties in obtaining such finance, the Government proposed to improve somewhat the priority position of new finance in administration.^73^

The following year, the Government—by now anticipating a significant need for ‘the refinancing of debt obtained during the boom’, including by larger companies^74^—proposed a more general restructuring moratorium, relaxing its prior focus on CVAs. The utility of CVAs for larger companies was limited by the fact that such arrangements cannot be used to affect the rights of secured creditors without their consent,^75^ and respondents to the first consultation had supported ‘the extension of the moratorium to other forms of restructuring, including informal (non-statutory) processes, and in particular schemes of arrangement implemented under the Companies Act’.^76^ The revised proposal was for a court-ordered moratorium (initially for three months) to provide a ‘breathing space, during which a restructuring could be negotiated and agreed’ by any means.^77^ No change was proposed to either the CVA or scheme procedures, which were ‘flexible and well regarded internationally’:^78^ the idea was simply to add the option for a moratorium to enable debtors to overcome coordination problems that could otherwise inhibit early restructuring.^79^

To qualify, a company would have to demonstrate a reasonable prospect of a compromise being agreed,^80^ and that it was ‘likely to have sufficient funds to carry on its business during the moratorium’:

Although the effect of the moratorium is to put pre-existing debts on hold, the company must be able to have the means to continue to trade during the moratorium, including meeting any new obligations that are incurred.^81^

Post-commencement liabilities would have super-priority if the moratorium were to be immediately followed by administration or liquidation proceedings, to give comfort to those trading with, or extending new finance to, a company under the moratorium.^82^ An insolvency practitioner would be appointed to monitor compliance with the qualifying conditions throughout.^83^ Directors would retain control rights but be subject to new restrictions and associated risks of personal liability.^84^

In 2011, the Government announced that it would not be rushing to implement this proposal. Consultation respondents had indicated that, ‘while the refinancing and restructuring of company debt remains a valid concern’, a moratorium was not as urgently required as had been expected, and respondents had raised other ‘difficult’ issues relevant to the efficacy of the moratorium, including the treatment of *ipso facto* clauses in contracts for supply.^85^ Respondents had also raised questions about the moratorium’s design, including in relation to the proposed qualifying conditions, as to which more detail was sought on which post-commencement liabilities would need to be paid (and attract priority to the extent unpaid), with concern expressed about the effects on secured lenders to the extent that this priority might reduce the value of a lender’s security.^86^ The Government pledged to work with stakeholders to revise the proposal and consider the additional issues that had been raised.

It was not until 2016 that a revised proposal appeared, and by now the Government’s tone had changed quite markedly. Whereas the initial proposals had been framed as an exercise in adding one or two elements, for which there was thought to be demand, to an otherwise well-functioning regime, the 2016 consultation was framed as an exercise in ensuring that the law was in line with evolving international ‘best practice’ in the design of corporate rescue frameworks.^87^ The idea that the UK was lagging behind appears to have been influenced by a fall in ranking in the 2015 ‘Doing Business: Resolving Insolvency’ assessment,^88^ after the methodology for that assessment was changed to incorporate a ‘law on the books’ component. UK law had been found not to exhibit all the features that economies were scored against, including whether the law enabled the continuation of ‘contracts essential to the debtor’s survival’ in insolvency proceedings.^89^ The 2016 proposal was for four major changes, one of which was to empower debtors to designate individual contracts for supply as ‘essential’ so as to prevent their termination or modification during an insolvency process.^90^ This would be a significant extension of the existing constraints on the termination and modification of contracts for the supply of utilities and IT services.^91^

The Government again proposed the introduction of a restructuring moratorium, and added a proposal for a ‘flexible restructuring plan’, in both cases contextualising its proposal by reference to the World Bank and IMF’s *Principles for Effective Insolvency and Creditor/Debtor Regimes*.^92^ The proposal for the moratorium was familiar, largely following the contours of the 2010 proposal, but with initiation by notice filing (filing documents with the court, rather than court order), and clarification that the requirement to have sufficient funds to carry on business meant that that the debtor was expected to be able to meet ‘current obligations as and when they fall due as well as any new obligations that are incurred’, whether to trade or financial creditors;^93^ pre-commencement arrears would be ‘frozen’.^94^

The proposal for the restructuring plan was new. The idea was for a procedure in which all creditors could be bound to a plan, including secured creditors (unlike in a CVA) and dissenting classes of creditor (unlike in a scheme of arrangement). The court would be empowered to declare a dissenting class bound if the prescribed majority in other classes had approved the plan, and the plan was:

in the best interests of the creditors as a whole, in that it recognises the economic rights of ‘in the money’ creditors and all other creditors are no worse off than they would be following liquidation.^95^

It would, however, only be appropriate to do so if the court was satisfied that the plan was ‘fair and equitable’.^96^ The Government’s thinking on what would be fair and equitable involved a combination of the requirement that no creditor be worse off than in liquidation, and two rules of priority—secured creditors were to be granted ‘absolute priority on repayment of debts’, and ‘junior creditors should not receive more on repayment than creditors more senior than them’.^97^ A fourth proposal, later dropped, related to the priority of post-commencement finance.

In 2018, the Government announced its intention to introduce a moratorium, constraints on rights to terminate or modify contracts for supply, and a restructuring plan procedure, as soon as parliamentary time permitted. As to the moratorium, the Government was satisfied that there was a case for introducing one ‘modelled on the same parameters as the administration moratorium’ to ‘reduce the costs and risks of restructuring’.^98^ It would be available by notice filing, and with similar qualifying conditions to those previously proposed, although the test of a ‘reasonable prospect’ of achieving a compromise would be tightened to require a rescue to be ‘more likely than not’.^99^ The most significant changes were that the Government proposed to restrict entry to companies not yet insolvent (to address concerns that the moratorium would be used to ‘delay an inevitable insolvency’, and encourage early use)^100^ and to reduce the initial length of the moratorium to 28 days (to strike ‘a good balance between allowing a company reasonable time to explore rescue options and temporarily suspending creditors’ rights to take enforcement action…’).^101^

The treatment of contracts for supply would be quite different to the 2016 proposal. Rather than enable debtors to designate particular contracts as essential, the Government would introduce a general constraint:

The Government will legislate to prohibit the enforcement of ‘termination clauses’ by a supplier in contracts for the supply of goods and services where the clause allows a contract to be terminated on the ground that one of the parties to the contract has entered formal insolvency.^102^

Suppliers would ‘retain the ability to terminate contracts on any other ground permitted by the contract’, other than grounds ‘connected with the debtor company’s financial position’.^103^ Where the constraint applied, suppliers would also be able to apply to court for relief on grounds of ‘undue financial hardship’, although this would be exceptional, the Government expecting that in most contexts the supplier would be sufficiently protected by the debtor’s obligation to pay being treated as an expense of the process.^104^

As to the restructuring plan, the Government was satisfied that there was a case for the introduction of a procedure which would ‘fill an existing gap in the company and insolvency frameworks’ by providing for cross-class cram down, and which could be used in combination with the moratorium to enable corporate rescue.^105^ The procedure would be modelled on the scheme procedure, involving an initial hearing at which class composition would be considered, voting on the proposed plan, and a second hearing on whether the plan should be sanctioned so as to become binding. There would be no financial condition for use of the procedure, in a bid to ‘reduce stigma and encourage earlier action on the part of directors’,^106^ but there would be various safeguards for creditors, including examination of class formation,^107^ a requirement that at least one impaired class approve a plan before the cross-class cram down power could be exercised,^108^ a comparison between the position of the dissenting class under the plan and in the next best alternative,^109^ the ‘basic principle that a dissenting class of creditors must be satisfied in full before a more junior class may receive any distribution’ but with flexibility to depart from this where ‘necessary to achieve the aims of the restructuring’ and ‘just and equitable in the circumstances’,^110^ and ‘absolute discretion’ for the court as to ‘whether or not to confirm a plan on just and equitable grounds’.^111^

### Implementing the Plan in a Pandemic

There was no time for the implementation of the Government’s plan prior to the onset of the pandemic, perhaps because so much governmental energy was taken up with managing the process of withdrawal from the European Union prior to ‘exit day’ on 31 January 2020. But by March 2020, an opportunity had arisen to implement the plan as part of a package of support offered to businesses in the pandemic. The Corporate Insolvency and Governance Bill was introduced on 20 May 2020 and received royal assent on 25 June 2020. CIGA contained all three elements of the pre-pandemic plan: to the Companies Act, CIGA added Part 26A, containing a new restructuring plan procedure to sit alongside the provision for schemes of arrangement in Part 26; to the Insolvency Act 1986, CIGA added Part A1, containing provision for a moratorium,^112^ and ss 233B-C, regulating contracts for the supply of goods and services to companies in proceedings under the Act, or for which a meeting had been summoned for the purpose of voting on a restructuring plan.

In relation to a debtor’s eligibility to use these measures, CIGA arguably improved on some aspects of the Government’s pre-pandemic thinking, at least as expressed in 2018. The moratorium is not restricted to businesses at a pre-insolvency stage: the CIGA test is that originally proposed, namely whether the debtor is or is likely to become unable to pay its debts.^113^ Conversely, while the 2018 proposal was that there would be no financial condition for use of the restructuring plan, CIGA restricts use of this procedure to companies that have encountered, or are likely to encounter, financial difficulties that are affecting, or will or may affect, the company’s ability to carry on business as a going concern, and for which the purpose of the proposed compromise or arrangement is to eliminate, reduce or prevent, or mitigate the effect of, those difficulties.^114^ Both of these changes appear desirable. But CIGA’s provisions also diverge in some *un*desirable ways from the pre-pandemic plan. In this section, I focus on some of the most striking of these divergences, beginning with the moratorium.

Much of what appears in new Part A1 is in line with the Government’s pre-pandemic plan for a moratorium. Part A1 makes provision for a moratorium, initially for 20 business days, obtainable by notice filing,^115^ which produces the same types of restrictions on enforcement as the administration moratorium does.^116^ Among the documents that must be filed to obtain the moratorium is a statement from the monitor^117^ that in their view ‘it is likely that a moratorium… would result in the rescue of the company as a going concern’.^118^ There is no requirement for certification that the company has sufficient funds to carry on business, but Part A1 produces a similar result by requiring the monitor to bring the moratorium to an end if they think^119^ that the company is unable to pay either ‘moratorium debts’ or ‘pre-moratorium debts for which the company does not have a payment holiday during the moratorium’ that have fallen due.^120^ Payment for supplies under a post-commencement contract for supply would be a ‘moratorium debt’;^121^ rent for a post-commencement period under a pre-commencement lease would be a ‘pre-moratorium debt for which the company does not have a payment holiday’.^122^

Remarkably, however, a liability under a pre-commencement ‘contract or other instrument involving financial services’ is also, unless and until the law is changed, a pre-moratorium debt for which the company does not have a payment holiday.^123^ A contract or other instrument involving financial services is defined to include a contract for the provision of a loan.^124^ Thus, the debtor enjoys no ‘breathing space’ from pre-commencement arrears owed to a lender.^125^ This is a major departure from the pre-pandemic plan, which was always premised on lenders being prevented from enforcing pre-commencement arrears.^126^ Moreover, there is no statutory constraint on the operation of rights of acceleration: all that the Act does (and this only as a result of amendments proposed by members of the House of Lords) is prevent the operation of such rights from producing super-priority for the lender for the accelerated amount in the event that the moratorium is followed by liquidation or administration.^127^ The overall result is that it is financial creditors, rather than the debtor, that are ‘in the driving seat’. Whatever the procedure is, it simply cannot be characterised (in its present form) as a tool for facilitating the restructuring of financial debt, which was of course the purpose of the original proposal.^128^

Were it not for a series of probing questions from members of the House of Lords in debate on the Bill, there would be little to explain this peculiar result. In the House of Commons, the moratorium was presented as affording ‘a company that is threatened with insolvency temporary respite from its creditors’^129^ (the pre-pandemic plan, but hardly consistent with the actual terms of the Bill), and little time was spent on the position of financial creditors, perhaps because the entire Bill ‘was rushed through… all stages: Second Reading, Committee and Third Reading—in four hours, 45 minutes’.^130^ In the House of Lords, however, there was greater concern about the use of the Bill to make permanent changes to the law (characterised as ‘cheeky’,^131^ and ‘opportunistic’^132^), and considerable concern about the particular position of financial creditors in the moratorium. Sustained questions on the latter point^133^ and a proposal to amend the Bill so that a debtor would enjoy a payment holiday in respect of liabilities under pre-commencement financial contracts (‘Amendment 20’, later withdrawn) pressed the Government into providing an explanation, which was eventually put in two ways: first, the privileged position of financial creditors was necessary to ensuring the stability of the financial system and the supply of credit *ex ante*; secondly, this position was necessary to ensure that financial services firms would be willing to extend new finance to debtors using the moratorium.^134^ There was no acknowledgment that fuller moratoria (on which the Government’s pre-pandemic plan was supposed to be modelled) were already available elsewhere in the insolvency framework.^135^

Criticism of the decision to insulate pre-commencement loans from the payment holiday might be said to be unfair, or at least overstated, given that the Act makes provision for the list of pre-moratorium debts for which the debtor does not enjoy a payment holiday to be amended, by the making of regulations.^136^ There is, therefore, an inbuilt mechanism for altering the position of financial creditors. One might, however, be sceptical about the prospects of change being made anytime soon, given the Government’s decision to explain the existing position as necessary to ensure financial stability, and the fact that making a change will not be a matter of simply ‘flicking a switch’—careful consideration will need to be given, for example, to the treatment of contractual rights of acceleration and set-off. In the meantime, the status quo is not a procedure that cannot be used at all, but rather a procedure that can be used to mandatorily extend the maturity of trade debt, and non-consensual creditor claims, whilst insulating financial contracts from this effect. This is a result that is difficult to defend.

The pre-pandemic plan for the regulation of contracts for the supply of goods and services was much less developed than the moratorium plan, with the proposal for a general constraint on suppliers’ termination rights only emerging in 2018. The 2018 plan did, however, make clear what the Government was interested in regulating, namely rights to terminate by reason of the debtor’s entry into an insolvency process, and what it was not interested in regulating, namely rights to terminate for reasons not linked to the commencement of an insolvency process or the debtor’s financial condition more generally.^137^ It is surprising, then, to find that new section 233B not only regulates rights to terminate by reason of the debtor entering into a procedure under the Insolvency Act 1986 (or for whom a meeting has been summoned for consideration of a restructuring plan),^138^ but also restrains the exercise of any entitlement to terminate because of a pre-commencement event—whether or not linked to the debtor’s financial condition.^139^ This is a much broader approach than that suggested by the pre-pandemic plan, and in other recent work^140^ I have suggested that whilst there may be a good case for introducing a constraint on the exercise of a right to terminate by reason of the debtor’s entry into a state-supplied procedure, that case does not extend to restraining rights to terminate that are not linked to the debtor’s financial condition.

It is unclear why section 233B was drafted so widely. It may be that the breadth of the provision reflects the Government’s particular concern to ensure continuity of supply during the pandemic.^141^ An alternative explanation is that the drafters, working at speed, simply borrowed from section 233A, a relatively recently added restraint on the exercise of rights to terminate contracts for the supply of ‘essential goods or services’, narrowly defined to mean the supply of utilities and IT-related services.^142^ Section 233A regulates not only rights to terminate such contracts by reason of the debtor’s entry into (certain) procedures,^143^ but also the exercise of a right to terminate by reason of a pre-commencement event.^144^ It also provides, however, that the supplier may insist on the office-holder personally guaranteeing the payment of charges for post-commencement supply as a condition of such supply.^145^ Section 233B does not contain this protection. In the debate on the Bill, the Government noted that a supplier to a company under the moratorium would be protected by the requirement to pay moratorium debts, and the super-priority afforded to such debts, to the extent unpaid, in a later liquidation or administration.^146^ But less was said about the position of the supplier in the (wide range of) other types of procedures in which section 233B applies. There, the supplier’s entitlement to be paid would appear to depend on the application of the expenses rules, which are a complex amalgam of statutory and judge-made law.^147^ This makes the position of the supplier unnecessarily precarious or, put another way, delivers the protection that section 233B aims at delivering (ensuring post-commencement continuity of supply) at unnecessary cost.^148^

The proposal for a new restructuring plan procedure was more fully developed, and much of new Part 26A of the Companies Act 2006 exhibits fidelity to the Government’s pre-pandemic thinking. Part 26A sets out a process for achieving a compromise or arrangement with creditors or any class thereof, or members or any class thereof, where the purpose of the compromise or arrangement is to ‘eliminate, reduce or prevent, or mitigate the effect of’ the debtor’s financial difficulties.^149^ The concepts of ‘compromise’ and ‘arrangement’ are understood to be used in broadly the same way as in the provisions for a scheme of arrangement in Part 26 of the Act,^150^ and the elements of the process for obtaining the court’s sanction have been modelled on Part 26.^151^ As expected, and unlike Part 26, Part 26A empowers the court to sanction a plan in circumstances in which the prescribed majority (75% or more in value of those present and voting^152^) of one or more classes have *not* approved the plan.

Section 901G contains two conditions that must be satisfied for the court to have power to sanction a plan in the presence of a dissenting class, although there is provision for these conditions to be varied by the making of regulations.^153^ The conditions are: first, that the court is satisfied that, if the compromise or arrangement were to be sanctioned, ‘none of the members of the dissenting class would be any worse off than they would be in the event of the relevant alternative’;^154^ secondly, that the compromise or arrangement has been agreed by the prescribed majority in value of a class of creditors or members ‘who would receive a payment, or have a genuine economic interest in the company, in the event of the relevant alternative’.^155^ Strikingly, there is no priority rule, in stark contrast to the pre-pandemic plan. It is clear from the drafting that satisfaction of the two conditions merely enlivens a discretion to sanction,^156^ but the statute is silent on the factors relevant to its exercise. As Mr Justice Snowden (as he then was) put it in *Virgin Active* with reference to the decision of Trower J in *DeepOcean I*:

Trower J first noted… that the statute gives little guidance on the factors that are relevant when the court is exercising its discretion to sanction a restructuring plan. I agree. Section 901G contains no express test or identification of any factors that should be taken into account, and leaves matters entirely at large….^157^.

The judge went on later in the judgment to add:

It is important to note that although it had been contemplated in the consultation process, an equivalent absolute priority rule [to that contained in the US Bankruptcy Code] was not enacted in any form as a principle for the exercise of the discretion in Part 26A.^158^

The best explanation for this departure from the plan is that it was simply too difficult to decide on the optimal formulation of the priority rule in an emergency context. The Government’s own thinking on this question had shifted between 2016 and 2018, and its 2018 proposal—to start with a requirement that dissenting classes be satisfied in full before a more junior class could receive value, but ‘inject flexibility’ so as to permit departures from this where necessary to achieve the aims of a restructuring, provided that this was ‘just and equitable’^159^—was not fully worked through. Rather than risk making a bad rule, the Government appears to have preferred to trust courts—already experienced with filling out the sparse statutory framework for schemes of arrangement—to develop the law, with a backstop of a statutory power to later amend section 901G. This is clear from the Explanatory Notes to CIGA,^160^ which convey the Government’s understanding that courts already enjoyed ‘absolute discretion’ when deciding to sanction a scheme of arrangement; that courts would enjoy the same discretion in relation to the decision to sanction a restructuring plan, and that this decision would import some consideration of whether the plan was ‘just and equitable’;^161^ and that courts would draw ‘where appropriate’ on schemes jurisprudence in articulating the factors relevant to the exercise of this discretion.^162^

There is of course no ‘absolute’ discretion in relation to schemes (at least if that is understood to mean discretion that is somehow unfettered—the discretion falls to be exercised in accordance with recognised principles), but even putting this to one side, it was a remarkable decision to leave the courts to do this work without the guidance that a priority rule would provide in cases in which all classes are better off than in the relevant alternative, but some more so than the dissenting class, particularly where that dissenting class is ‘in the money’. Plainly, the schemes jurisprudence cannot supply an answer, because there is no power to sanction a scheme without the assent of the requisite majority of each class that is made a party to it. Indeed, this—the essential difference between a scheme and a restructuring plan—means that caution must be exercised when transplanting the schemes jurisprudence to the plan context, as courts have since acknowledged. The principle in the schemes case law that the court ‘will be slow to differ from the meeting’^163^ is premised on the scheme having the support of the requisite majority of those at that meeting. Again, as Mr Justice Snowden put it in *Virgin Active*, with reference to the prior decision of Trower J in *DeepOcean I*:

Trower J… explained that the court should not have the same reluctance to differ from the vote at a class meeting when considering whether to exercise the power to cram down as it would have when considering whether to sanction a scheme under Part 26. Again, I agree. Under Part 26, the fact that the court is considering whether to sanction a scheme presupposes that the majority in each class has voted in favour of the scheme, and the issue is whether the court should nevertheless differ from the will of the majority and refuse to sanction it. Under Part 26A, the use of the cram-down presupposes that a class has either failed to approve the plan by the necessary majority… or contains a majority which has positively expressed disapproval by voting against the plan…^164^

… [w]hilst a rationality test can be applied when considering whether to sanction a scheme under Part 26 which has been approved by a majority in each relevant class, the same test cannot necessarily be applied in the same way when the court is considering whether to exercise the power under section 901G to sanction a Part 26A plan against the views expressed by a dissenting class.^165^

In omitting a priority rule, then, the legislature left difficult work—work in determining questions of distributional entitlement—to the courts.

The courts are unquestionably rising to this task. In a series of admirably clear judgments, judges have, *inter alia*, rejected the presence of a freestanding test of whether the plan is ‘just and equitable’ as devoid of content,^166^ identified where the schemes jurisprudence remains salient and where it is less likely to be so,^167^ and begun to articulate the principles relevant to the exercise of the discretion to sanction in the presence of a dissenting class. As to the latter, the emerging case law points to a concept of ‘fair distribution of the benefits of the restructuring’^168^ that draws on the case law on the statutory test of ‘unfair prejudice’^169^ in company voluntary arrangements. In CVAs, creditors are grouped together (recall, however, the debtor’s inability to affect the entitlement of secured and preferential creditors without consent),^170^ rather than separated into classes. In recognition of this, courts interrogate differential treatment across the group, as well as confirming that creditors are receiving more than they would in the relevant alternative, in deciding whether a CVA is ‘unfairly prejudicial’. Differential treatment is not fatal, but ‘must be justified’.^171^ In *Re Houst Ltd*, Zacaroli J held (in the context of a decision to sanction a plan in the presence of a dissenting ‘in the money’ class) that section 901G similarly requires the court ‘to see whether the plan provides for differences in treatment of creditors inter se and, if so, whether the differences are justified’.^172^ In the plan context—where a plan could bind secured and preferential creditors as well as unsecureds—a relevant reference point would be ‘the treatment of the creditors in the relevant alternative’, and in particular ‘whether the priority, as among different creditor groups, applicable in the relevant alternative is reflected in the distributions under the plan’.^173^ A departure would not be fatal—after all, the legislature had chosen not to include the priority rule proposed in 2018^174^—but would call for justification. This points to an emerging (flexible) priority principle.^175^

Despite the courts’ evident capacity, there are reasons to be doubtful of the legislature’s decision to delegate this work. There is an obvious transition cost associated with this mode of rule-making: it will likely require a good number more cases, decided over (at least) several more years, before the law is regarded as sufficiently settled to be predictable.^176^ Once it is sufficiently predictable, the question will be whether a superior rule might have emerged through the ordinary legislative process. This is not inconceivable: after all, analogical reasoning is ‘usually not the most systematic or reliable way to evaluate the effects of laws’,^177^ and the effect of this particular law is quite profound, given that it may be used to bind not only ‘in the money’ financial creditors (who can adjust to the rules once settled) but also other, less well-adjusting classes of creditor, without the consent of the majority. If a superior rule is available, it is unlikely to now be written, notwithstanding the formal power of the legislature to rewrite the conditions in section 901G: once settled, a change to the law will likely be opposed by the relevant professional bodies, for whom the law will have become familiar. Finally, a rule produced in this way will not enjoy the same legitimacy as one produced through the legislative process. This is a non-trivial point, given that the question being determined is a question of distributional entitlement.

### The Overall Result: A Lost Opportunity

Individual components of CIGA’s permanent measures can of course be changed, and it may be that some of the difficulties identified above are corrected sooner rather than later, although I have expressed some scepticism about the likelihood of this. What will be harder to undo, however, is the structural decision to implement the pre-pandemic plan by simply tacking the proposed measures on to the existing (pre-pandemic) framework. In the House of Lords debate on the Bill, Lord Mendelsohn was doubtful as to whether the plan would have been implemented in this way outside of the emergency (‘the permanent provisions were consulted on, although in their previous form they were never going to be implemented in such a piecemeal fashion’) but concluded ‘we are where we are, and the Government are going to do this whatever we say’.^178^ He focused on the mechanisms that would be put in place to review CIGA’s operation and to make changes in the light of this evidence. But such *ex post* review and amendment cannot fully substitute for an *ex ante* assessment of how a proposal for insolvency law reform can be situated most effectively within the existing legal framework, and of what changes are required to ensure that the overall framework remains cogent.

The pre-pandemic law was already a complicated mix of procedures, each with a different scope and producing different effects in relation to the position of directors and the treatment of executory contracts on commencement,^179^ and in relation to the vulnerability of pre-commencement transactions to avoidance.^180^ These procedures could be used selectively (to compromise some classes of liability but not others)^181^ and flexibly (including by combining procedures, and by using procedures in a ‘pre-packaged’ way). A small business, for example, might restructure unsecured debts through a company voluntary arrangement, but might also achieve the same result through a pre-packaged administration in which the business is sold on commencement to a new company owned by the owner-manager of the old, with some creditors left behind in the old company,^182^ thanks to an interpretation of the statute that enables administrators to exercise their powers before taking proposals to creditors.^183^ Understanding each procedure requires a grasp of the statutory regime, as interpreted by the courts, and of the ways in which the statute has been augmented by judge-made law. The liability of directors, for example, is governed by rules in the Insolvency Act 1986 (including those imposing liability for wrongful and fraudulent trading^184^) and the Company Directors Disqualification Act 1986 (into which the Government inserted a new route to personal liability in 2015^185^), as well as by the judge-made ‘rule in *West Mercia*’, which was recently the subject of a 130-page Supreme Court decision in which members of the Court disagreed on important facets of the rule.^186^ This complexity meant that the Government’s March 2020 promise to suspend the wrongful trading rule^187^ had a more limited effect than its communications suggested.^188^

All of this remains true after CIGA, but the menu for corporate debtors has become even more complex, with two new procedures on offer, and a new constraint in most procedures on the exercise of rights to terminate by reason of the debtor’s entry into the procedure—but only in relation to contracts for the supply of goods and services. It is of course not inherently objectionable to offer debtors a menu of procedures, each with different features; indeed, there may be good reasons to do so.^189^ In the English case, however, it is not clear that differences between procedures and gaps between them are the result of a deliberative exercise, rather than the inadvertent result of a decision to layer new options over old, without regard to overall fit and cogency. In the restructuring context, for example, consider the following: was it desirable to introduce constraints on the termination of contracts for the supply of goods and services whilst leaving the statute largely silent on the subject of unexpired leases? What advantages does the new moratorium offer compared with a restructuring through a pre-packaged administration sale to a new company owned by stakeholders in the old, and what is the intended relationship between these two processes?^190^ Does it make sense to formally require a debtor to enter administration or liquidation proceedings to access most of the transaction avoidance provisions of the Insolvency Act 1986, whilst leaving judge-made rules that can be applied to produce a similar result potentially applicable outside these proceedings?^191^ Can the Government’s vision of early initiation of a restructuring process by the debtor-in-possession be reconciled with the plethora of personal liability risks for the directors of insolvent companies, to which the Government itself added in 2015? These are just some of the questions that might have been asked if the Government’s pre-pandemic plan had been pursued in the ordinary way.

## Could We Have Done Better?

Those involved in the passage of the Bill were working under very demanding conditions. Difficult design decisions had to be made quickly if the opportunity to implement reforms that were expected to be useful—both in the emergency and more generally—was not to be squandered. Yet, there is much to regret in the overall result. CIGA departed from the pre-pandemic plan in material and undesirable ways, and its rushed implementation meant that an opportunity to consider how best to fit this plan to the pre-existing framework was lost. Overall, CIGA appears to exemplify the risks of reforming insolvency laws in a crisis, rather than the opportunities that a crisis affords to the insolvency policymaker.

Could this result have been avoided? One obvious lesson of the CIGA experience is that a policymaker is not well-prepared to seize the opportunity afforded by a crisis merely by identifying the ways in which the insolvency regime might usefully expand the protection it offers to debtors vis-à-vis their creditors and counterparties. The essential next step is to map the ways that this enhanced protection might be delivered and, if the preference is to deliver this by adding new options to the menu (rather than by a more ambitious rationalisation), to carefully appraise the relationship between the new options and the existing menu, or (put another way), to test ‘fit’. It is difficult to see how policymakers can make credible claims about the expected net benefit of adding to the menu without robustly assessing fit. One way to do so would be to identify the kinds of case that the policymaker expects will benefit from treatment under the expanded menu, and then (in an impact assessment) trace through the expected effects of that treatment; such an exercise may reveal gaps in legislative provision, or inconsistencies, or other barriers to realising the policymaker’s goal. This exercise may be particularly important where part of the inspiration for the change is evidence of a departure from international ‘best practice’ (rather than evidence of the operation of the existing framework); ‘problems of fit’ may be particularly prevalent here.

Of course, no amount of preparation can fully protect a plan from being distorted by enactment in crisis conditions. The most obvious protection against the risk that in-crisis rule-making will produce an undesirable result is to provide that the changes made in the crisis expire after a prescribed period (say, 2 years) unless Parliament acts to procure their extension (a ‘sunset’ clause).^192^ In fact, this approach was suggested for CIGA by members of both the House of Commons and the House of Lords, but rejected by the Government on the basis that CIGA’s permanent measures had ‘not just been developed in the short time since Covid-19 first appeared’, but rather ‘been the subject of a considerable period of consultation and engagement…’^193^—an answer that obscures, of course, the fact that the text of the Bill departed in material ways from the pre-pandemic plan. The moratorium has not been well received by stakeholders,^194^ and it seems plausible to posit that this measure might have been permitted to expire had a sunset clause been included—thus enabling the Government to ‘reset’ its approach.^195^ It must be conceded that if Part 26A had been enacted in the same form as in CIGA, but with the addition of a sunset clause, the measure would likely have been made permanent: the restructuring plan procedure has been much more enthusiastically received than CIGA’s other measures.^196^ But this thought experiment seems highly artificial: in reality, the design of Part 26A—and in particular the decision to task courts with filling out the legislative framework over time—does not appear to be compatible with a sunset clause strategy. Having set the law on this path, the best that can now be done is to critically assess what was lost by this decision, as well as what was gained, with a view to informing analogous reform exercises in future.

## Conclusion

The onset of a crisis may appear to provide a significant opportunity to an insolvency policymaker, but the pursuit of that opportunity in crisis conditions will be fraught with risk. In the UK, the onset of the pandemic appeared to provide an opportunity to implement long-planned restructuring law reform. The outcome, however, was permanent legislation that deviated from the pre-pandemic plan in material and undesirable ways. The overall result is not an insolvency regime that will postpone the closure of unviable businesses at creditor expense, nor one in which creditors will be forced into protracted restructuring negotiations where their interests would be best served by a rapid sale of the business or assets to a third party: the limited scope of the new moratorium in Part A1 of the Insolvency Act 1986, combined with the absence of any built-in moratorium in the provision for restructuring plans in Part 26A of the Companies Act 2006, means that debtors have only been given very limited ‘breathing space’. But CIGA has produced other distortions, and introduced unnecessary costs, and set the law on a particular path (more choice from an even more bewildering menu) from which it will be difficult now to turn. Overall, there is much that can and should be learned from the CIGA story for approaches to restructuring law reform in future crises.

## Footnotes

1 Posner (1997).

2 OECD, ‘Insolvency and debt overhang following the COVID-19 outbreak: Assessment of risks and policy responses’, Policy Brief, 27 November 2020, p 17; see also Djankov (2009) (identifying bankruptcy reforms as ‘one of the reforms that are easier to do in crisis’); Cirmizi et al. (2012), p 197.

3 See Cirmizi et al. (2012) on this trend in the post-global financial crisis period.

4 See van Zwieten et al. (2021).

5 Ibid.; see also Eidenmueller and van Zwieten (2015).

6 OECD (2020). On the adoption of impact assessment systems in Europe, and variation in the design of these systems, see Dunlop and Radaelli (2016).

7 House of Lords Select Committee on the Constitution, 3^rd^ Report of Session 2021-22, ‘COVID-19 and the use and scrutiny of emergency powers’, HL Paper 15, 10 June 2021, pp 2, 7.

8 Organised groups that are economically affected by the shock may be best positioned to exert influence on the legislative process: Junk et al. (2022).

9 OECD, ‘Regulatory quality and COVID-19: The use of regulatory management tools in a time of crisis’, Policy Brief, 30 September 2020.

10 Of the permanent changes made by the Act: see ‘Corporate Insolvency and Governance Bill: Impact Assessment’, 21 April 2020. Two reports were subsequently delivered by P. Walton and L. Jacobs: ‘Corporate Insolvency and Governance Act 2020 – Interim report March 2022’, and ‘Corporate Insolvency and Governance Act 2020 – Final Evaluation Report November 2022’. Both are available at www.gov.uk/government/publications/corporate-insolvency-and-governance-act-2020-evaluation-reports (last accessed 14 March 2023).

11 See Schedules 10 and 11 of CIGA.

12 By the ‘suspension’ of the wrongful trading rule: see ss 12-13 of CIGA and below, text to nn. 41 and 188.

13 Known prior to the Insolvency Acts of 1985-86 as ‘receivership and management’: see van Zwieten (2018), ch. 10.

14 For the exceptions, where it remains possible for an administrative receiver to be appointed today, see Insolvency Act 1986 (UK), Pt. III, Ch. IV.

15 Insolvency Act 1986, s. 9.

16 See van Zwieten (2018) para. 11.04 and (as to the receiver’s duties) para 10.48 et seq.

17 See Enterprise Act 2002, s. 250(1); Enterprise Act 2002 (Commencement No. 4 and Transitional Provisions and Savings) Order 2003/2093.

18 Insolvency Act 1986, Sch. B1, paras 14, 22.

19 Insolvency Act 1986, Sch. B1, para. 3.

20 Department of Trade and Industry (2001).

21 See Part I of the Insolvency Act 1986. The CVA procedure can be used in or outside of administration, but the arrangement cannot affect the right of a secured creditor, or the distributional entitlement of a preferential creditor, without their consent (s. 4).

22 Including by using administration to execute (perhaps on a ‘pre-packaged’ basis) a sale to a new company owned by stakeholders in the old, leaving those ‘out of the money’ behind, and adding a scheme of arrangement to bind dissentient secured creditors. See Paterson (2018); *Re DeepOcean I UK Ltd* [2021] B.C.C. 483, [50].

23 House of Commons Library, ‘Corporate insolvency framework: proposed major reforms’, Briefing Paper CBP8291, 5 December 2019, p 4.

24 See Part 26A of the Companies Act 2006.

25 Insolvency Act 1986 Part A1 (moratorium) and ss 233B-C (contracts for supply).

26 See van Zwieten (2018), para. 7-14; K. van Zwieten, ‘Contracts for the supply of goods and services’, Working Paper, 2022.

27 Insolvency Service, ‘Encouraging company rescue – a consultation’, June 2009. The proposal was refined in Insolvency Service, ‘Proposals for a restructuring moratorium – a consultation’, July 2010.

28 Insolvency Service, ‘A review of the corporate insolvency framework: a consultation on options for reform’, May 2016. As with the consultation documents immediately above, this was accompanied by an impact assessment.

29 Department for Business, Energy & Industrial Strategy, ‘Insolvency and corporate governance: Government response’, 26 August 2018.

30 Department for Business, Energy and Industrial Strategy, Department of Health and Social Care, Public Health England, Prime Minister’s Office, 10 Downing Street, and The Right Hon Sir Alok Sharma KCMG MP, ‘Business Secretary’s statement on coronavirus (COVID-19): 28 March 2020’.

31 Hansard, HC Deb (3 June 2020), vol. 676, col. 892.

32 See CIGA s. 3 and Sch. 4 (relaxing the conditions for using the moratorium), and s. 15 (modifying the constraints on suppliers to temporarily exclude small suppliers).

33 Department for Business, Energy & Industrial Strategy, ‘Grant Funding Schemes: Small Business Grant Fund and Retail, Hospitality and Leisure Grant Fund, Guidance for Local Authorities’, March 2020.

34 Chancellor Rishi Sunak, Budget Speech 2020, 11 March 2020, available at www.gov.uk/government/speeches/budget-speech-2020 (last accessed 14 March 2023).

35 The Coronavirus Job Retention Scheme. A scheme for the self-employed (the Self-Employment Income Support Scheme) followed.

36 Value-added tax, normally charged on most goods and services.

37 Chancellor Rishi Sunak, ‘Updated statement on coronavirus’, speech given at Downing Street, 20 March 2020, available at www.gov.uk/government/speeches/the-chancellor-rishi-sunak-provides-an-updated-statement-on-coronavirus (last accessed 14 March 2023).

38 See ‘Budget Speech 2020’, n. 34 above, and ibid.

39 The Covid Corporate Financing Facility, to be operated by the Bank of England on behalf of the Treasury. See letter from Andrew Bailey, Governor of the Bank of England, to Chancellor Rishi Sunak, 17 March 2020, available at www.bankofengland.co.uk/-/media/boe/files/letter/2020/letter-from-the-governor-to-the-chancellor-march-2020.pdf (last accessed 14 March 2023); Bank of England, ‘Joint HM Treasury and Bank of England Covid Corporate Financing Facility (CCFF) – Market Notice, 18 March 2020’, available at www.bankofengland.co.uk/markets/market-notices/2020/ccff-market-notice-march-2020 (last accessed 14 March 2023).

40 Ministry of Housing, Communities & Local Government, Department for Business, Energy & Industrial Strategy, HM Treasury, The Rt Hon Alok Sharma KCMG MP, The Rt Hon Rishi Sunak MP, and The Rt Hon Robert Jenrick MP, ‘Press Release: Extra protection for businesses with ban on evictions for commercial tenants who miss rent payments’, 23 March 2020.

41 ‘Business Secretary’s statement on coronavirus (COVID-19): 28 March 2020’, n. 30 above.

42 Ibid.

43 Department for Business, Energy & Industrial Strategy, Ministry of Housing, Communities & Local Government, The Rt Hon Robert Jenrick MP, and The Rt Hon Sir Alok Sharma KCMG MP, ‘Press Release: New measures to protect UK high street from aggressive rent collection and closure’, 23 April 2020.

44 Ibid.

45 ‘Chancellor of the Exchequer, Rishi Sunak on COVID19 Response’, speech delivered on 17 March 2020, transcript available at www.gov.uk/government/speeches/chancellor-of-the-exchequer-rishi-sunak-on-covid19-response (last accessed 14 March 2023).

46 Although the Government did encourage commercial tenants to ‘pay what they can’: ‘New measures to protect UK high street from aggressive rent collection and closure’, n. 43 above.

47 HM Treasury, ‘Covid-19 and bank lending’, 25 March 2020, signed by Chancellor Rishi Sunak, Andrew Bailey (Governor of the Bank of England) and Chris Woolard (Chief Executive of the Financial Conduct Authority), available at www.bankofengland.co.uk/-/media/boe/files/letter/2020/covid-19-and-bank-lending.pdf (last accessed 14 March 2023).

48 Consistently with this, see Bank of England, ‘The Bank of England’s Term Funding Scheme with additional incentives for SMEs will open to drawings on 15 April 2020’, 6 April 2020, available at www.bankofengland.co.uk/news/2020/april/the-tfsme-will-open-to-drawings-on-april-15-2020 (last accessed 14 March 2023).

49 Bank of England, ‘Opening statement by the Governor and Andrew Bailey’, 11 March 2020, available at www.bankofengland.co.uk/-/media/boe/files/news/2020/march/opening-remarks-march11-2020.pdf (last accessed 14 March 2023).

50 ‘The Bank of England’s Term Funding Scheme’, n. 48 above.

51 See ‘Opening Statement’, n. 49 above.

52 Bank of England (2022).

53 Department for Business, Energy & Industrial Strategy, ‘COVID-19 loan guarantee schemes repayment data as at March 2022, updated 5 September 2022’.

54 1,201,081 of 1,278,570 facilities reported to be on schedule: ibid.

55 HM Treasury, ‘Bounce Back Loan borrowers can delay repayments by extra six months’, 8 February 2021. The option was exercised for 28% of Bounce Back Loans: ‘COVID-19 loan guarantee schemes repayment data as at March 2022, updated 5 September 2022’, n. 53 above.

56 Department for Business, Energy & Industrial Strategy, ‘Apply for a coronavirus Bounce Back Loan’, 27 April 2020.

57 National Audit Office (2021), para. 1.3.

58 Ibid., p 13.

59 The British Property Federation estimated that, as at 30 June 2021, £7.5 billion of commercial rent would be in arrears: Department for Levelling Up, Housing and Communities, ‘Supporting businesses with commercial rent debts: policy statement’, 4 August 2021. Later, the Commercial Rent (Coronavirus) Act 2022 introduced a bespoke arbitration regime for the treatment of pandemic commercial rent arrears.

60 In particular, the Government announcement promising the legislation (‘Business Secretary’s Statement’, n. 30 above), the parliamentary debates on the Bill (as recorded in Hansard), and the Explanatory Notes prepared by the Department for Business, Energy & Industrial Strategy to assist the reader.

61 In the House of Commons, the Bill had its second reading and third reading on the same day. See further, House of Commons Library Research Briefing, ‘Expedited legislation’, available at www.commonslibrary.parliament.uk/research-briefings/sn04974/ (last accessed 14 March 2023).

62 See, for example, the Explanatory Notes prepared to accompany the Bill’s introduction to the House of Commons (‘Corporate Insolvency and Governance Bill’ Explanatory Notes, 20 May 2020), at paras 78-96 (‘Fast-track legislation’); Hansard, HL Deb (9 June 2020), vol. 803, cols 1673 and 1725; Hansard, HC Deb (3 June 2020), vol. 676, cols 890 and 892.

63 Insolvency Service, ‘Encouraging company rescue – a consultation’, n. 27 above.

64 Ibid., para. 25.

65 Ibid., para. 26.

66 Ibid., para. 30.

67 Ibid., para. 4.

68 See Insolvency Act 1986, s. 1.

69 Insolvency Service, ‘Encouraging company rescue – a consultation’, n. 27 above, para. 4.

70 At the time that the CVA procedure was introduced (Insolvency Acts of 1985-1986), a moratorium was only available by the company entering into liquidation or administration. The Insolvency Act 2000 introduced a moratorium for small companies, available by notice filing: see s. 1A and Sch. A1 of the Insolvency Act 1986 as added by the 2000 Act. These provisions were repealed by CIGA, as proposed by the Government in 2018 (see n. 99 below).

71 Insolvency Service, ‘Encouraging company rescue – a consultation’, n. 27 above, para. 40.

72 Provided that in the court’s view the moratorium would be in the interests of creditors as a whole, ibid., para. 46.

73 Ibid., para. 54 et seq.

74 Insolvency Service, ‘Proposals for a restructuring moratorium – a consultation’, n. 27 above, para. 2.2.

75 See n. 21 above, and ‘Proposals for a restructuring moratorium – a consultation’, n. 27 above, Annex C (Impact Assessment), p 29.

76 Insolvency Service, ‘Encouraging company rescue: summary of responses’, November 2009, para. 24.

77 Insolvency Service, ‘Proposals for a restructuring moratorium – a consultation’, n. 27 above, p 5.

78 Ibid., para. 2.3.

79 Ibid., Annex C (Impact Assessment), pp 28, 31; see also para. 2.7.

80 Ibid., para. 3.16, subject to paras 3.19-3.21.

81 Ibid., para. 3.17. These criteria appear to have been closely modelled on those in Sch. A1 of the Insolvency Act 1986 (relating to the small company moratorium): see paras 6-7 of Sch. A1 (now repealed).

82 Ibid., para. 4.25. More specifically, the unpaid debt would have priority over the expenses of the administration or liquidation (see n. 4 on p 21), such expenses already having priority (to the extent that unencumbered assets are insufficient) over the entitlement of a floating charge holder to look to their security for satisfaction (see further van Zwieten (2018), para. 8-35).

83 Ibid., para. 4.12.

84 Ibid., paras 4.9, 4.20.

85 ‘Written Ministerial Statements – Wednesday 11 May 2011, Business Innovation and Skills, Restructuring Moratorium (Consultation)’, Hansard, HC Deb (11 May 2011), col. 37WS. On *ipso facto* clauses, the Government reported: ‘There have been suggestions that a greater impact might be achieved by the restructuring moratorium were it also to tackle issues such as termination clauses (whereby suppliers can cancel essential contracts and threaten the viability of company’s (sic) rescue plans)…’.

86 In particular, through the erosion of the value of a floating charge (as to which, see n. 82 above): Insolvency Service, ‘Proposals for a restructuring moratorium: summary of responses’, May 2011, paras 11, 42-45, 91-98.

87 Insolvency Service, ‘A review of the corporate insolvency framework: a consultation on options for reform’, n. 28 above, p 5.

88 See van Zwieten 2022 Working Paper, n. 26 above, and ibid., para. 2.3.

89 See World Bank, ‘Doing Business Archive’, ‘Resolving insolvency: strength of insolvency framework index’, available at https://archive.doingbusiness.org (last accessed 14 March 2023).

90 Insolvency Service, ‘A review of the corporate insolvency framework: a consultation on options for reform’, n. 28 above, Ch. 8.

91 See ss 233-233A of the Insolvency Act 1986. The restraint on termination and modification was added by the Insolvency (Protection of Essential Supplies) Order 2015/989.

92 Insolvency Service, ‘A review of the corporate insolvency framework: a consultation on options for reform’, n. 28 above, paras 7.1 and 9.7.

93 Ibid., paras 7.22 and 7.11. This would suggest that interest falling due for payment in a post-commencement period would be payable, while pre-commencement arrears would not be. Nothing was said about the treatment of contractual clauses enabling the acceleration of a liability.

94 Ibid., para. 7.11.

95 Ibid., para. 9.20.

96 Ibid., para. 9.27.

97 Ibid., para. 9.32.

98 Department for Business, Energy & Industrial Strategy, ‘Insolvency and corporate governance: Government response’, n. 29 above, paras 5.9-5.10.

99 Ibid., para. 5.31. The existing small company moratorium available in connection with a proposal for a CVA would be repealed: para. 5.14.

100 Ibid., para. 5.28; compare with para. 7.18 in the 2016 proposal (n. 28 above). It may be that this change was also influenced by the European Commission’s Proposal for a Directive of the European Parliament and of the Council on preventive restructuring frameworks, second chance and measures to increase the efficiency of restructuring, insolvency and discharge procedures and amending Directive 2012/30/EU COM(2016) 723 final, published on 22 November 2016 (after the 2016 proposal was published, but also after the Brexit referendum, which was held on 23 June 2016). I am grateful to Riz Mokal for drawing my attention to this point.

101 Ibid., para. 5.49.

102 Ibid., para. 5.97.

103 Ibid., para. 5.99.

104 Ibid., para. 5.105 et seq.

105 Ibid., para. 5.123.

106 Ibid., para. 5.131.

107 Drawing on schemes jurisprudence: ibid., para. 5.151.

108 Ibid., para. 5.167.

109 Ibid., paras 5.114 and 5.169 et seq.

110 Ibid., paras 5.164-5.165.

111 Ibid., para. 5.166, see also para. 5.152.

112 For ‘eligible companies’, see ss A1, A2, Sch. ZA1.

113 S. A6(1)(d), and s. A54(1) (defining inability to pay debts for a registered company as having the same meaning as in Part 4 of the Insolvency Act 1986).

114 Section 901A(1), (2), (3) of the Companies Act 2006 (‘Conditions A and B’). These go to the jurisdiction of the court to sanction a plan and are considered at the initial (convening a meeting) stage: see *Re Virgin Atlantic Airways Ltd* [2020] B.C.C. 997; *Re Amicus Finance plc* [2022] B.C.C. 18.

115 Sections A3, A7, A9 of the Insolvency Act 1986 and, as to the possible forms of extension, ss A10-A15.

116 See s. A21 (moratorium) and, by way of comparison, para. 43 of Schedule B1 (administration). The crystallisation of a floating charge is also restricted during the moratorium: ss A22, A52.

117 Who is an officer of the court: s. A34.

118 S. A6(1)(e), in line with the 2018 proposal (above, text to n. 99). As to ongoing monitoring, see ss A35, A38(1)(a).

119 On the standard by which a monitor would be judged, see *Re Minor Hotel Group* [2022] Bus. L.R. 908.

120 Section A38(1)(d); see also s. A42. See also ss A10, A11 and A13 on the conditions for the extension of the moratorium.

121 See ss A54(1) and A53(2). As to the meaning of ‘debt or other liability’, see s. A54(1), (2).

122 See ss A54(1) and A53(1)(b), read together with s. A18(3)(c). Section A53(4) provides that the Secretary of State may, by regulations, alter the definitions of ‘moratorium debt’ and ‘pre-moratorium debt’.

123 S. A18(3)(f). Wages or salary arising under a contract of employment, and redundancy payments, are also excluded: ss A18(3)(d), (e) and A18(7).

124 Section A18(7) and Schedule ZA2, para. 2; *Re Minor Hotel Group* (n. 119 above) [13].

125 ‘The exclusion of finance debts from the “payment holiday” effects of a moratorium is somewhat surprising: but it is the clear meaning of the amended Act’: *Re Minor Hotel Group* ibid. [14] per Sir Alastair Norris.

126 See text to nn. 81 and 94 above. Whilst nothing was said in 2016 and 2018 about the treatment of acceleration clauses, the premise was clearly that lenders would be forced to wait: this was the reason for the decision to shorten the moratorium in 2018 (above, text to n. 101; see also para. 5.68 in Department for Business, Energy & Industrial Strategy, ‘Insolvency and corporate governance: Government response’, n. 29 above); see also, consistently with this, paras 7.26, 7.27 and 7.46 in the 2016 proposal, ‘A review of the corporate insolvency framework: a consultation on options for reform’, n. 28 above.

127 See s. 174A of the Insolvency Act 1986, Explanatory Notes to CIGA, para. 134, and Payne (2021), cited in *Re Minor Hotel Group*, n. 119 above, at [15].

128 Consistently with this, in a 2022 survey of insolvency practitioners, over half (53%, n=96) ‘felt that the Moratorium was either not at all effective or somewhat ineffective in providing companies a period of protection to seek advice, negotiate with creditors and agree plans for rescue’, and the treatment of financial contracts was reported to be ‘seen by some as a major reason why it may be of limited use in practice’: ‘Corporate Insolvency and Governance Act 2020 – Final Evaluation Report’ (n. 10 above), noting that the procedure had been used 40 times between 26 June 2020 and 30 September 2022. See also Paterson (2021), p 322.

129 Alok Sharma, Hansard, HC Deb (3 June 2020), vol. 676, col. 893.

130 Lord Blencathra, Hansard, HL Deb (9 June 2020), vol. 803, col. 1696.

131 Baroness Neville-Rolfe, Hansard, HL Deb (9 June 2020), vol. 803, col. 1680, confessing to doing the same when pulling law reform ‘from the famous Whitehall drawer’ when a previous crisis provided an opportunity.

132 Ibid., Baroness Barker, col. 1714; see also on the same day Lord Hodgson, col. 1688, Lord Palmer, cols 1697-1698, Lord Fox, col. 1721, Baroness Kramer, col. 1681, Baroness Bowles, col. 1678.

133 Including from Baroness Bowles (Hansard, HL Deb (16 June 2020), vol. 803, col. 2075), Baroness Drake (col. 2115 on the same day, describing the Government’s reasoning as ‘difficult to comprehend’), and Lord Fox, proposing to sunset the moratorium provisions after two years (cols 2138 and 2140 on the same day). Sunsetting had also been proposed in committee stage in the House of Commons (see Hansard, HC Deb (3 June 2020), vol. 676, col. 958).

134 Hansard, HL Deb (23 June 2020), vol. 804, col. 151, and Hansard, HL Deb (16 June 2020), vol. 803, col. 2125. See also the Explanatory Notes to CIGA, para. 134, and *Re Minor Hotel Group* (n. 119 above) [16].

135 It may be objected that in administration, directors are displaced from exercising management powers (these are entrusted to the administrator, an insolvency practitioner: see Sch. 1, Insolvency Act 1986), while the moratorium leaves the debtor ‘in possession’. But directors can be permitted to continue to exercise management powers in administration (see Sch. B1, para. 63), and moreover administration can be ‘pre-packaged’ so that a sale is arranged (including to a new company owned by stakeholders in the old: n. 22 above, and below, text to n. 182) before directors are displaced. Additionally, the previous moratorium for small companies proposing a CVA (repealed by CIGA: see n. 70 above) was a ‘debtor in possession’ process in the sense that management powers remained with directors rather than being transferred to an insolvency practitioner.

136 See n. 122 above. Such regulations are subject to the affirmative resolution procedure: s. A53(5).

137 Above, text to n. 103.

138 S. 233B(1)-(3), subject to (5) and (6).

139 S. 233B(4), subject to (5). The entitlement may not be exercised during the ‘insolvency period’, as defined in s. 233B(8). A supplier can apply to the court for relief on the basis of ‘hardship’ (see s. 233B(5)(c) and above, text to n. 104), which borrows from s. 233A of the Act.

140 See van Zwieten 2022 Working Paper, n. 26 above.

141 See above, text to n. 42.

142 See ss 233A(7), 233(3), and above, text to n. 91.

143 See s. 233A(1), (8)(a), (b).

144 Section 233A(1), (8)(c).

145 Section 233A(3), (5), and as to who is the office-holder, (9).

146 Hansard, HL Deb (9 June 2020), vol. 803, col. 1726.

147 See van Zwieten (2018), at para. 11-112. In this regard, it is noteworthy that CIGA temporarily exempted small suppliers from s. 233B (n. 32 above,), and that in the House of Lords, Lord Vaux proposed a permanent exemption (Amendment 51, later withdrawn) on the basis that ‘having to supply with uncertainty of payment… will be disproportionate at any time, pandemic or no pandemic’: Hansard, HL Deb (16 June 2020), vol. 803, cols 2157-2158.

148 See further van Zwieten 2022 Working Paper, n. 26 above.

149 Section 901A(3) of the Companies Act 2006, and see n. 114 above and accompanying text.

150 *Re Virgin Atlantic Airways Ltd*, n. 114 above, at [38], [46] (per Trower J); *Re Gategroup Guarantee Ltd* [2021] B.C.C. 549, [141] et seq.; *Re Amicus Finance plc* [2022] B.C.C. 18, [73]; *Re Smile Telecoms Holding Limited* [2022] B.C.C. 808, [25] et seq.

151 Explanatory Notes to CIGA, para. 15; see further Judiciary of England and Wales, ‘Practice Statement (Companies: Schemes of Arrangement under Part 26 and Part 26A of the Companies Act 2020)’, and as to differences between the two, see *Virgin Atlantic* ibid., [44] per Trower J (convening judgment) and [41]-[42] per Snowden J (sanctioning judgment).

152 See s. 901F(1) of the Companies Act 2006.

153 See s. 901G(6)-(7) of the Companies Act 2006, and as to the similar provision in Part A1 of the Insolvency Act 1986 (for the moratorium), see above, n. 122. In both cases, such regulations are subject to the affirmative resolution procedure, which requires parliamentary approval.

154 Section 901G(3) of the Companies Act 2006. The relevant alternative is ‘whatever the court considers would be most likely to occur in relation to the company if the compromise or arrangement were not sanctioned’: s. 901G(4); see also *Re DeepOcean I*, n. 22 above, [29]-[38]; *Re Hurricane Energy Plc* [2021] B.C.C. 989.

155 Section 901G(5), requiring agreement by a number representing 75% in value of the class present and voting either in person or by proxy at the meeting summoned by the court (see also s. 901F).

156 The court ‘may’ sanction the compromise or arrangement: see *DeepOcean I*, n. 22 above, [42].

157 *Re Virgin Active Holdings Limited* [2022] 1 All E.R. (Comm) 1023, [213]; see also *DeepOcean I*, ibid., at [44], *Re Houst* [2022] B.C.C. 1143, [22].

158 *Virgin Active*, ibid., [289].

159 Above, text to n. 110.

160 As to the relevance of these in interpreting CIGA, see *Virgin Active*, n. 157 above, [215].

161 Explanatory Notes to CIGA, paras 15, 190-192; see also Hansard, HL Deb (9 June 2020), vol. 803, cols 1684, 1726.

162 Explanatory Notes to CIGA, para. 16; see also *Hurricane Energy*, n. 154 above, at [31].

163 *Re Telewest Communications plc (No 2)* [2005] B.C.C. 36, [22] (decision of David Richards J).

164 *Virgin Active,* n. 157 above, [214].

165 Ibid., [222]; see also *Hurricane Energy*, n. 154 above, at [40]-[48].

166 *Virgin Active*, n. 157 above, [219]; *Houst*, n. 157 above, [23].

167 See *Virgin Active,* ibid., [228] et seq., distinguishing between differential treatment of some but not all creditors who are out of the money, and differential treatment of those in the money; *Hurricane Energy*, n. 154 above, [44] et seq.; *DeepOcean I*, n. 22 above, [44] et seq.; *Houst*, n. 157 above, [24]-[27].

168 *DeepOcean I*, ibid., [63] per Trower J; *Houst*, n. 157 above, [29]*; Re E D & F Man Holdings Limited v In the Matter of the Companies Act 2006* [2022] EWHC 687 (Ch), [58]; see also *Virgin Active*, n. 157 above, [228]-[256]. *Houst* and *Virgin Active* draw on two papers by Professor Riz Mokal: Mokal (2020) and Mokal (2021).

169 See Insolvency Act 1986, s. 6(1).

170 See above, text to nn. 75 and 21.

171 *Discovery (Northampton) Ltd v Debenhams Retail Ltd* [2020] B.C.C. 9, [12], [102] et seq., drawn on in *DeepOcean I*, n. 22 above (see [62] et seq.); *Lazari Properties 2 Ltd v New Look Retailers Ltd* [2021] Bus. L.R. 915.

172 *Houst,* n. 157 above, [29].

173 Ibid., [30]; see also Mokal (2021).

174 *Houst*, ibid., the judge endorsing the analysis of Professor Sarah Paterson (in a 2022 Working Paper, ‘Judicial discretion in Part 26A restructuring plan procedures’) that the omission of a priority rule had to be taken to be deliberate.

175 See also *Re Smile Telecoms Holdings Limited* [2021] EWHC 685 (Ch), [38]; *E D & F Man Holdings*, n. 168 above, [58] (‘there is no reason to consider that the benefits of the restructuring plan, what is sometimes called the restructuring surplus, is being shared in an inequitable manner which does not reflect plan creditors existing rights. The plan itself respects the current ranking of creditor claims’), [64].

176 Of course, there may also be uncertainty after the introduction of a statutory provision, depending on the way it is designed (see generally Kaplow (1992)). I thank Riz Mokal for pushing me on this point.

177 Sunstein (1993), p 758.

178 Hansard, HL Deb (16 June 2020), vol. 803, col. 2137.

179 Consider, e.g., the differing effects of commencement of liquidation and administration on directors (van Zwieten (2018), at paras 5-24, 11-94); the historical unavailability of s. 213 (fraudulent trading) and s. 214 (wrongful trading) of the Insolvency Act 1986 in administration (see the same text at para. 11-17); the power of disclaimer, presently confined to liquidation (same text at para. 6-26); the evolving jurisprudence on the applicability of the so-called ‘*Lundy Granite* principle’ in administration (same text at para. 11-112); the scope of the anti-deprivation rule (same text at ch. 7), compared with s. 233A of the Insolvency Act 1986 (only applicable where a company enters administration or a CVA takes effect in relation to it: s. 233A(1)).

180 Sections 238 and 239 of the Insolvency Act 1986 only being available in liquidation and administration, such that they are unavailable when the company uses the CVA procedure or scheme of arrangement procedure outside of liquidation or administration. However, a transaction that the directors authorise in breach of duty, where that breach is known to the counterparty, may be void at general law (*GHLM Trading Ltd v Maroo* [2012] 2 B.C.L.C. 369, [171]), and the content of directors’ duties is affected by insolvency (see the cases cited below, n. 186).

181 Paterson and Walters (2022).

182 Such ‘connected party pre-packs’ have attracted criticism and latterly have been made subject to increased regulation: see Administration (Restrictions on Disposal etc. to Connected Persons) Regulations 2021/427.

183 *Re Transbus International Ltd (in liquidation)* [2004] 1 W.L.R. 2654.

184 See Insolvency Act 1986, ss 213, 214, 246ZA, 246ZB.

185 See s. 15A of the Company Directors Disqualification Act 1986 and, critiquing the reform, van Zwieten (2020a).

186 See *BTI 2014 LLC v Sequana SA* [2022] 3 W.L.R. 709; see also *Stanford International Bank Ltd (in liquidation) v HSBC Plc* [2023] 2 W.L.R. 79.

187 Above, text to n. 41.

188 See van Zwieten (2020b).

189 Canipek et al. (2019).

190 During the passage of the Bill, some members of the House of Lords raised questions about the regulation of connected party pre-packs (prompting the Government to amend the Bill to revive a regulatory power (see s. 8 of CIGA), later exercised: n. 182 above), but on the question of the intended relationship between the moratorium and pre-packaged administration, the discussion clarified only the Government’s expectation that the former not be used as a precursor to the latter (see Hansard, HL Deb (23 June 2020), vol. 804, cols 134 and 150).

191 Above, n. 180.

192 I am grateful to Paul Davies for pushing me on this point.

193 Hansard, HL Deb (9 June 2020), vol. 803, col. 1730.

194 Above, n. 128.

195 On s. 233B, the Final Evaluation Report on CIGA (‘Corporate Insolvency and Governance Act 2020 – Final Evaluation Report November 2022’, n. 10 above) concluded: ‘[I]t remains too early to assess fully whether the policy objectives … have been satisfied, though the early signs are positive’.

196 Ibid., section 4.2.
